# The Effect of Material Properties on the Perceived Shape of Three-Dimensional Objects

**DOI:** 10.1177/2041669520982317

**Published:** 2020-12-26

**Authors:** Masakazu Ohara, Juno Kim, Kowa Koida

**Affiliations:** Department of Computer Science and Engineering, Toyohashi University of Technology, Toyohashi, Japan; Sensory Processes Research Laboratory, School of Optometry and Vision Science, University of New South Wales, Sydney, Australia; Department of Computer Science and Engineering, Toyohashi University of Technology, Toyohashi, Japan; Electronics-Inspired Interdisciplinary Research Institute, Toyohashi University of Technology, Toyohashi, Japan

**Keywords:** 3D perception, motion, shape, surfaces/materials

## Abstract

Perceiving the shape of three-dimensional objects is essential for interacting with them in daily life. If objects are constructed from different materials, can the human visual system accurately estimate their three-dimensional shape? We varied the thickness, motion, opacity, and specularity of globally convex objects rendered in a photorealistic environment. These objects were presented under either dynamic or static viewing condition. Observers rated the overall convexity of these objects along the depth axis. Our results show that observers perceived solid transparent objects as flatter than the same objects rendered with opaque reflectance properties. Regional variation in local root-mean-square image contrast was shown to provide information that is predictive of perceived surface convexity.

Images result from the complex interplay between illumination, surface materials, and three-dimensional (3D) shape. We perceptually untangle these complex patterns of image structure to visually infer the shape of 3D objects and their reflectance properties. However, the perceptual experience of surfaces with material properties is not always correct, and errors in perception can occur in either the recovery of a surface’s material properties or 3D shape (Belhumeur et al., 1999; Khang et al., 2007; Marlow et al., 2015; Mooney & Anderson, 2014; Nefs et al., 2006; Nishida & Shinya, 1998; Todd et al., 2015; Vangorp et al., 2007; Wijntjes & Pont, 2010). Previous work has shown that the perceived 3D shape of opaque objects tends to be underestimated—that is, the angle between the perceived surface normal and the line of sight (Bernhard et al., 2016; De Haan et al., 1995; Koenderink & van Doorn, 1992; Todd et al., 2004). We considered how the perception of surface convexity depends on the surface optics of different materials.

Most natural surfaces have a certain degree of specularity that contributes to the perception of shape. Specular reflectance generates luminance variations in images that highly depend on the structure of the surrounding light field (Norman et al., 1995, 2004; Olkkonen & Brainard, 2011). Early work has shown that these specular reflections of the surrounding light field contribute to the perception of shape (Fleming et al., 2004). More recently, Mooney and Anderson (2014) showed that the perceived convexity of surfaces tended to be overestimated by different magnitudes depending on the simulated material composition. They found that the convexity of purely diffuse surfaces (i.e., using Lambertian shading) viewed in depth was significantly overestimated compared with ground truth. They also showed that adding sharp specular reflections using a bidirectional reflectance distribution function increased perceived convexity in excess of the surface’s true curvature. These findings suggest that glossier surfaces tend to be perceived as bumpier than diffuse surfaces, and diffuse surfaces tend to be perceived as bumpier than what they are. However, increase in bumpiness of opaque surfaces can also increase the complexity and number of specular reflections and perceived surface gloss (Ho et al., 2008; Marlow et al., 2012; Wijntjes & Pont, 2010). It is possible that these complex changes in specular and diffuse shading might also influence the perception of 3D shape.

Another surface property that might influence perceived shape is the refractive nature of transparent objects. Transparent objects are abundant in daily life (e.g., water droplets, ice, crystal). It is important to accurately perceive their 3D structure so we may interact with them. For example, we effortlessly can pick up ice from within a glass cup. These thick transparent objects have different degrees of refractive power, depending on their shape and material composition (Fleming, Jäkel, et al., 2011; Schlüter & Faul, 2014). Do the refractive properties of transparent surfaces influence the perception of their 3D shape?

Previous research suggests that human observers are not able to accurately estimate the refractive index (RI) of transparent objects. One study showed that perceptual judgments of transparency loosely correspond to the physical refractive indices of test objects (Fleming, Jäkel, et al., 2011). The researchers proposed that perception of transparency was estimated based on the background distortion seen through transparent objects (Fleming, 2014; Fleming, Jäkel et al., 2011; Todd & Norman, 2019). Another study proposed that background distortions alone are not sufficient for perceiving RI because they depend on both the shape and distance of the object from the background (Schlüter & Faul, 2014). Rather than observers matching internal experiences of refractivity, these researchers found that observers tended to match surfaces directly based on similarity in image cues: specular reflections and the distortion field. Further studies have used gauge figure tasks to estimate variations in perceived surface slant (i.e., surface curvature) and found that the 3D shape of objects with semiopaque reflectance properties tends to be perceptually underestimated (Chowdhury et al., 2017; Schlüter & Faul, 2019). These interactions between perceived shape and material properties suggest that the perception of both shape and materials depends on similar sources of image-based information.

How might the visual system determine whether distortions of the light field are generated by refraction or reflection? Both shiny metallic and transparent objects reflect images of the surrounding light field that are distorted by the 3D shape of the object. A research study found that inverting an image of a solid refractive globally convex object could transform its appearance to shiny metal (Kim & Marlow, 2016). The key requirement for this material inversion effect is some consistency in the perception of shape between upright and inverted images. Normally, the inversion of the light field would only occur for metallic objects when they are concave rather than convex. The perception of a convex solid glass object with light field inversion suggests the distortions themselves provide cues to both shape and material composition. In a recent article, Tamura et al. (2018) showed that moving surfaces generate dynamic motion cues that can improve observer performance in differentiating between opaque glossy and transparent refractive materials. These perspective motion cues are likely to help observers to also infer the 3D shape of moving objects.

Here, we examined the effect of varying material properties and motion constraints on the perception of a globally convex object’s 3D shape. Previous work studied the perception of curvature from diffuse and specular shading (Kim & Anderson, 2010; Mooney & Anderson, 2014; Motoyoshi et al., 2007), but perceived shape of diffusely shaded objects may differ from other surfaces with semiopaque properties (Chowdhury et al., 2017; Fleming, Holtmann-Rice, et al., 2011; Schlüter & Faul, 2014). Therefore, we systematically varied the simulated material composition of objects from refractive to reflective, with different amounts of specular reflectance (Figure 1). Observer estimates of surface convexity were then obtained to determine whether there were any variations in perceived shape from ground truth and between different material classes. This allowed us to tease apart the effects of material properties and surface motion on perceived convexity, and whether there are image cues that are predictive of the perception of shape.

**Figure 1.**
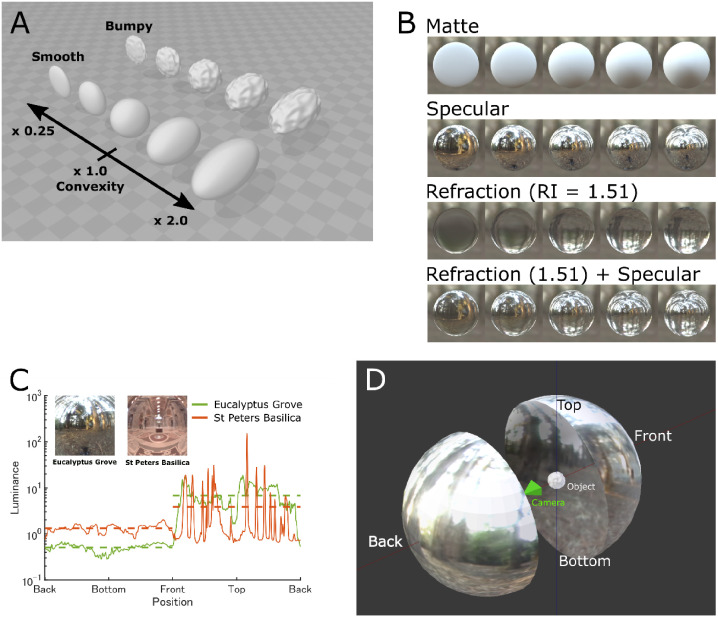
The 3D stimulus objects and environments. A: Object meshes were either smooth or bumpy and had one of five convexities in depth (range × 0.25 to × 2.0). The object at ×1.0 corresponds to the purely spherical surface. B: Sample renderings of smooth surfaces with different material properties which looking from the center of the object. Index of refraction for transparent objects was 1.51, equivalent to Crown glass. Matte surfaces were rendered with only diffuse reflectance. Specular conditions were rendered with only specular reflectance. C: Luminance distribution along the angle of elevation of two light fields used in this study. Luminance along the azimuth was averaged. Inset images show the central square region of the light fields. Horizontal dotted lines show the mean luminance either at top or bottom part of the light fields. D: Layout of the object, camera for rendering, and light field. A movie example can be found in Supplemental Movie.

## Method

### Observers

Eight adult observers participated in the experiment, all of whom had normal or corrected-to-normal vision. The participants ranged in age from 22 to 44 years. Informed consent was obtained from all participants. Procedures were approved by the Toyohashi University of Technology ethics committee. All research was performed in accordance with the relevant guidelines and regulations.

### Stimuli

All the stimuli were rendered using the open-source rendering package Blender 3D (Ver. 2.78b). The 3D geometry was created in Blender 3D by taking an initial Ico Sphere with 20,480 triangle faces and 10,242 vertices (i.e., subdivisions = 6). Bumpy surface relief was created by generating a Clouds texture (Size = 0.25, Depth = 0, Nabla = 0.03). This texture was applied to the geometry as a height map using the Displace Modifier (Midlevel = 0.5, Strength = 0.2). The Ico Sphere shape and Clouds texture and Displace Modifier are included in Blender 3D. The number of bumps along the circumference was 5–12, and its peak amplitude was 5% to 7% of the diameter of the object, based on Fourier power analyses of the radial distance of the object silhouette image (Figure 1A). All the stimuli were rendered with Cycles Render in Blender 3D. Cycles Render is a physically based ray tracing render engine designed for high-quality animation. The objects were rendered after they were multiplicatively scaled in depth over five levels (0.25, 0.5, 1.0, 1.5, and 2.0; hereafter referred to as *convexity*).

We rendered images without tone mapping; the intensity of the specular highlights was limited by setting the exposure, and any values exceeding the dynamic range of our standard RGB rendering were set to 255. The monitor’s calibrated gamma for displaying these images was 2.2 (mean absolute error was 5.7%, measured by a color and luminance meter CS-200, Konica Minolta).

We used two light fields for rendering, either the Eucalyptus Grove or the St Peters Basilica (Figure 1C; source: http://www.pauldebevec.com/Probes/). There were four surface material properties: Matte, Specular, Refraction (RI = 1.51), and 50% Refraction (RI = 1.51) + 50% Specular (Figure 1B). We also used three sizes of 3D objects that were small, normal, or large in diameter: length of 0.5, 1.0, and 2.0 Blender Units (BU), with the corresponding display size of 6.2, 12.4, and 24.8 deg, respectively. These were made by scaling the object’s shape multiplicatively equally along the X, Y, and Z directions.

The camera position was fixed to 10 BU away from the 3D object’s center. Light fields were represented on a sphere with infinite diameter. Objects were oscillated sinusoidally along the horizontal axis at 1.0 Hz (amplitude = 2 BU, corresponding display size: 24.8 deg) for 5 seconds beginning from the center and moving rightwards. All movie presentations of horizontal object oscillation were rendered using a custom Python script executed in the Blender environment. All movies were rendered at 60 fps.

### Procedure

Visual stimuli were presented using custom psychophysical software called Psymat (ver. 0.35; http://juno3d.com/software/) running on a PC (OS: Windows 10 Pro, CPU: Intel Core i7-6700, Graphic card: GeForce GTX 960).

Observers were seated, and their head fixed by a chin rest. Stimuli were presented on an LCD flat-panel display (HP E242) situated 30 cm in front of the observers. Stimulus size was 28.6 × 28.6 cm, 51 × 51 deg, 800 × 800 pixels including the object and background of the light fields, and screen area other than the stimulus movie was midgray (red = green = blue = 0.5). Observers were given a minute at the start of the experiment to practice a small number of randomly presented trials, before moving on to the formal testing session. In a block of trials, observers were shown stimulus movies for 5 seconds with both eyes open, and then they were asked to attend to the curvature of objects in depth. Participants were specifically asked to rate how flat or elongated the objects appeared on a global scale.

Following each 5-second movie presentation, the scene was replaced with a metric scale for observer ratings. The vertical bar on the left was used to indicate the length, and the oval on the right corresponds to the profile of the 3D object when it was viewed from above. The rating bar and 2D outline were modulated simultaneously when the observer pressed either the UP/DOWN arrow key to respectively increase or decrease the matched profile length. The observer was asked to report the 3D object’s appearance as more elongated (like a rugby ball) or flatter (like a pancake). No feedback on their response accuracy was provided.

Once the observer was satisfied with their match, they pressed the space bar to log the response and advance to the next trial. The order of stimuli was randomized within a block of 80 conditions (4 materials × 5 convexity levels × 2 bump levels × 1 size in diameter × 2 light fields). Observers performed three repeats for each stimulus condition. On another day, observers participated in the same experiment, but the size of the objects was modified to small or large size with the order of object sizes randomly intermingled between blocks of trials.

### Static Presentations

To identify the effect of static cues on the perception of surface convexity, we presented the same stimuli statically in a follow-up experiment to determine whether there were any declines in perceptive convexity. In this follow-up experimental session, stimulus objects did not move and were presented statically at either the center, left, or right side of the stimuli locus traversed in the dynamic viewing experiment. A total of 240 randomized trials were presented to each observer (4 materials × 5 convexity levels × 2 bump levels × 2 light probes × 3 stimuli positions).

### Statistical Analysis

The results were analyzed using MATLAB (Ver. R2016b). We tested for a main effect of light probe, bump level, physical convexity, and material property. The analysis of variance (ANOVA) treated physical convexity as a continuous factor but was treated as a categorical factor in post hoc testing. The other factors were treated as categorical factors in all analyses. We also tested for interaction effects between physical convexity and material property.

## Results

### Effect of Dynamic Material Properties on Shape Perception

**Figure 2.**
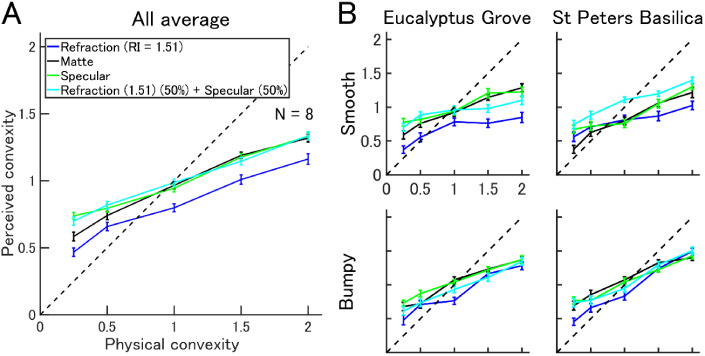
Perceived convexity for different material properties and lighting conditions for middle-sized dynamic stimuli. A: Plots show mean perceived convexity as a function of physical convexity of objects with different simulated material properties. The dashed line shows perception equating to ground truth. B: Results were shown separately by different illumination conditions and different surface bumpiness. Error bar shows standard error of mean of all trials. RI = refractive index.

In our first task, observers estimated the perceived convexity of moving objects with different physical convexity, bumpiness, and material properties rendered under two light fields (Figure 2). Perceived convexity increased significantly with physical convexity—four-way ANOVA, main effect of physical convexity: *F*(1, 1919) = 1445.23, *p* < .001, partial η^2^ = 0.4332. Generally, objects with convexity larger than 1.0 (i.e., greater than spherical) were underestimated relative to ground truth, and objects with convexity smaller than 1.0 (i.e., flatter than spherical) were overestimated relative to ground truth (dashed line). Thus, the slope of perceived convexity with respect to physical convexity was smaller than the ground truth (Slope = 0.377, linear regression of all material properties). Perceived convexity also differed depending on material properties—four-way ANOVA, main effect of material properties: *F*(3, 1919) = 16.91, *p* < .001, partial η^2^ = 0.0262. Across all convexity levels, objects that were purely refractive were perceived significantly flatter than specular objects of identical convexity (post hoc test, Tukey honest significance test [HSD], *p* < .001, within all other materials). The average difference in perceived convexity between the purely refractive and the other materials was approximately 0.166. A complete description of the statistics is shown in Table S1 in Supplemental Material.

The Refraction (50%) + Specular (50%) surface was perceived as comparable in convexity to the specular surface. Presumably, this occurred because the specular reflection component dominated observer’s estimates of convexity. The flattest (convexity = 0.25) matte object was perceived significantly flatter than the specular object of equivalent convexity (post hoc test, Tukey HSD, *p* = .018).

The effect of changing material properties on perceived convexity was generally consistent across different light fields and surface bumpiness (Figure 2B). In all four subplots, perceived convexity significantly increased with physical convexity (post hoc test, Tukey HSD, *p* < .001, convexity = 0.25 vs. convexity = 2.0). Again, across all convexity levels in the Eucalyptus Grove light field, smooth objects that were purely refractive were perceived significantly flatter than other materials of identical convexity (post hoc test, Tukey HSD, *p* < .001, within all other materials), but not for all stimulus and viewing conditions (result shown in Table S1 in Supplemental Material). There were no significant differences between the flattest matte object and the specular object of equivalent flatness (post hoc test, Tukey HSD, *p* = .786 [Grove–Smooth], *p* = 1 [Grove–Bumpy], *p* = 0.057 [St Peters–Smooth], *p* = 1 [St Peters–Bumpy]). Results were reproduced for different object sizes (Figure S1 and Table S2 in Supplemental Material).

### Effect of Static Perspective Cues on Perceived Convexity

Moving objects provide several cues to infer their convexity, including motion cues and viewing perspective in addition to image cues (e.g., specular distortions). We performed another task in attempt to isolate these cues, but this time the stimuli were static images of the object placed at the center, extreme left, or extreme right of the image plane. We raise the hypothesis that looking at the sides (the left/right condition) might provide strong enough cues to shape, and then the result might be worse in the center condition and better in left/right condition.

**Figure 3.**
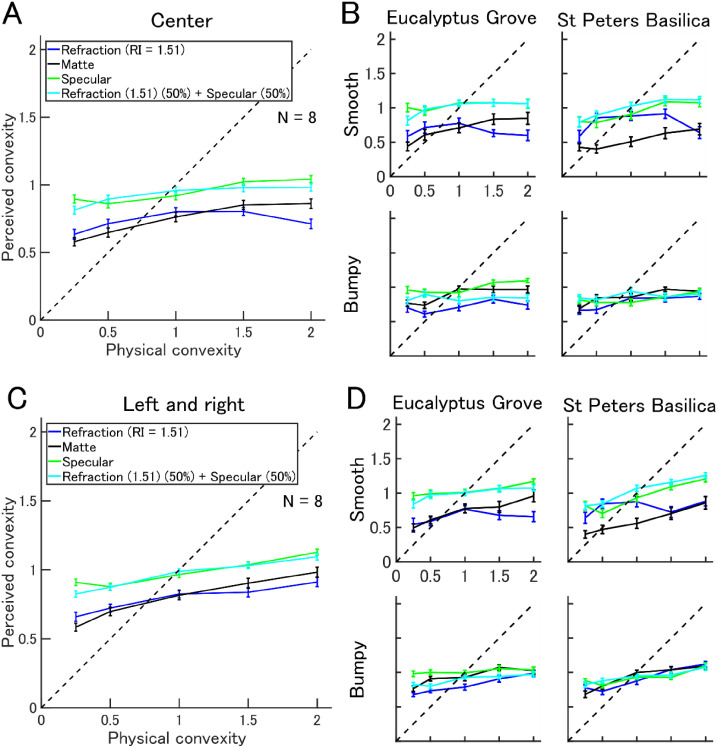
Effect of material properties on perceived convexity for static stimuli. Format is same as in Figure 2. A and B: Results for the object placed on center. C and D: Results for the object placed on either left or right. RI = refractive index.

Results are shown separately for the three positions of the object (Figure 3). Because left and right positions induced similar results, they were averaged. We performed a five-way ANOVA on these data to test the main effects of object position × light probe × bump level × convexity × material property. Slopes were smaller than in the motion condition, and the slopes of the center condition were smaller than left/right conditions (slope, center = 0.102, left/right = 0.161: linear regression across all material properties). The difference between positions was not significant (five-way ANOVA, main effect of positions: *p* = .35). Again, we found the effect of material properties on perceived convexity (*p* < .001, details shown in Table S3 Supplemental Material). Perceived convexity increased as physical convexity increased (main effect of convexity). The flattest (convexity = 0.25) matte object was perceived significantly flatter than the specular object of equivalent convexity (post hoc test, Tukey HSD, both center and the left/right: *p* < .001). Across all convexity levels, objects that were purely refractive or purely matte were perceived significantly flatter than objects with specular reflectance and identical convexity (post hoc test, Tukey HSD: *p* < .001; Figure 3A and C).

These results are generally consistent across surface bumpiness and different light fields (Figure 3B and D). Across all convexity levels, objects that were purely refractive were perceived significantly flatter than specular objects of identical convexity except for bumpy objects in the St Peters Basilica for both the center and left/right conditions (post hoc test, Tukey HSD, *p* < .001).

It is possible these data could be explained by a learning effect because the same observers participated in the task with static images after the dynamic image task. The aforementioned hypothesis was rejected by the results; poor performance for both center and left/right, even if it had a learning effect.

The results together suggest that material properties generate static image cues for modulating the estimation of shape. However, in the absence of motion cues, the linear relationship between perceived and physical convexity was small. The decline in the slope of the relationship in the static viewing task does not appear to be due to increased uncertainty of object shape, because the error bars obtained for our perceptual estimates are similar to those of dynamic viewing tasks where a steeper slope was observed. These results suggest that dynamic and static cues contribute to the perception of shape, and these cues depend on the surface optics of different materials.

### Image Analysis

We found that the surface properties of objects affect the perception of their shape. How do the image cues produced by the interaction of the light field with different material surfaces evoke different perception of shape? We examined a potential explanation for our observed results.

Curvature involves a change in surface orientation relative to the observer in depth that generates shading critical for shape perception (Fleming, Holtmann-Rice, et al., 2011; Ramachandran, 1988; Todd & Mingolla, 1983). This shading generates a variation in local contrast in image luminance that can be used to inversely estimate surface convexity. For example, consider a side view of either a flat or elongated (highly curved) object with matte surface properties embedded within an anisotropic light field (e.g., Eucalyptus Grove). The image of the flat surface will have a shallow diffuse shading gradient and lower local contrast at almost all visible surface regions—and thus, its luminance variation will be globally small. In contradistinction, the image of the elongated surface will have a steeper diffuse shading gradient and higher local contrast at the position where surface normals are oriented more obliquely to the primary lighting direction from above causing the regional variation in local contrast to be larger for the elongated surface compared with the flatter surface.

Curvature also generates variation in the scaling of texture gradients (Todd & Thaler, 2010), which influences the rate of change in luminance over finite regions of image space. Similar compression of environmental reflections occurs across specular surfaces (Fleming et al., 2004). The strength of local contrast cues generated by specular edge contours is known to be important for the perception of gloss (Kim, Tan, et al., 2016), which might also contribute to the perception of shape. It is possible that the perception of convexity can be modeled by computing variations in *local* contrast across the surface image. Therefore, we analyzed whether perceived surface convexity could be explained by the variations in local root-mean-square (RMS) contrast of the stimulus images by following formula:

$$\mathrm{RMS}\;\mathrm{contrast}\;=\;\sqrt{\frac{1}{N}\sum_{n=1}^{N}|{\left|x_{n}\right|}^{2}|}$$

where *x* is pixel luminance, and *N* is number of pixels for analysis.

**Figure 4.**
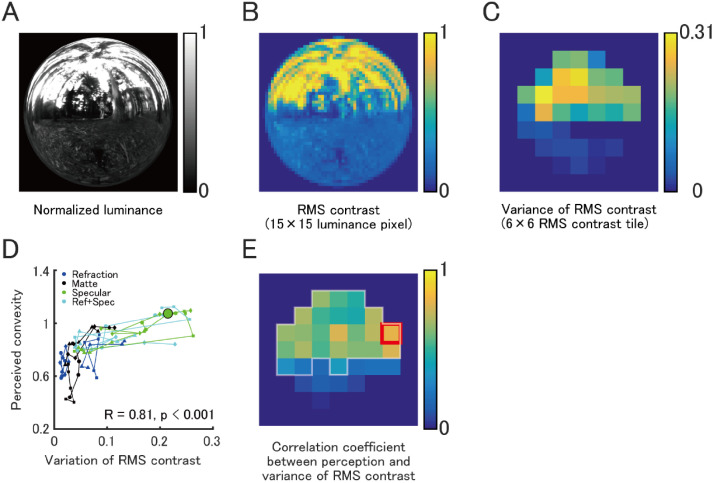
Correlation between local RMS contrast variability and perceived convexity. A: Normalized luminance image of an example stimulus. B: Local RMS contrasts over finite image regions defined within 15 × 15 pixels were computed. C: The variance of RMS contrast within the regions of 6 × 6 RMS contrast areas was computed. D: Correlation plots between the variance of RMS contrast of the region with highest correlation (denoted by red square in E) and perceived convexity. Color of symbols shows surface properties. Shape of symbol indicates the light probe and stimulus shape (circle: Grove–Smooth, diamond: Grove–Bumpy, square: St Peters–Smooth, triangle: St Peters–Bumpy). Symbols connected by lines indicate five levels of stimulus convexity (E). The Spearman’s rank correlation coefficient was calculated separately for each variance of RMS contrast. White outlined areas showed significant correlation (*p* < .05). RMS = root-mean-square.

We computed local RMS contrast over finite image regions defined within 15 × 15 pixel (1.0 × 1.0 deg) square tiles (Figure 4). Then, we calculated the variance of RMS contrast over regions defined within 6 × 6 tiles. Thus, each variance was computed over 90 × 90 pixels (6.1 × 6.1 deg) of the stimulus image.

To assess the validity of our model, we computed the correlation coefficient between the variance of local RMS contrasts and perceived convexity (Figure 4D and E). The data for perceived convexity used here were for the condition with static stimuli presented at the center of the display. The large green symbol in Figure 4D indicates the data from the smooth sphere (convexity = 1) in the Eucalyptus Grove light probe (Figure 4A to C). Figure 4E shows the distribution of the correlation coefficients (Spearman’s R value) for each analyzed image area. Significant correlations were observed for the upper half of the image (significant correlation, *p* < .05). Highest correlation was found for right side of the image (*R* = 0.81, red outline in Figure 4E). A similar conclusion was obtained for different region size for calculating RMS contrast and tile size for calculating the variance (Figure S2 in Supplemental Material). These results suggest that perceived convexity can be explained by variance of local RMS contrasts at different parts of the image.

## Discussion

We found that an object’s material properties (specular, matte, or refractive) biased its perceived convexity. Refractive and matte objects were overall perceived as flatter than the same geometry rendered with specular reflectance. Combined material properties (refractive 50% + specular 50%) were perceived as having similar shape to purely specular surfaces, indicating that the specular component dominated percepts of shape. Variation in local RMS contrast around the upper half image region was shown to provide predictive leverage in estimating perceived surface convexity.

Consistent with previous research, specular objects were perceived as having greater relief height than matte objects of equivalent convexity (Kim, Khuu, et al., 2016; Mooney & Anderson, 2014; Nefs et al., 2006). The perceived convexity of static matte objects was lower than dynamically moving objects. Motion generated percepts of shape that were closer to veridical than the most oblique left-right static views (Wendt et al., 2010; Wendt & Faul, 2019). This was particularly the case for matte surfaces, which generated stronger percepts of convexity in moving simulations compared with static presentations. These findings suggest that motion provides useful optic flow information above and beyond the shape of the bounding contour alone. We also found that the perceived convexities for both matte and purely refractive objects were lower than for objects with any specular reflection. However, differences in perceived convexity were smaller for bumpy surfaces compared with smooth surfaces as indicated by the difference in the displacement of material response curves in our experiments.

Across all materials, we find that flat surfaces were generally overestimated in convexity, and elongated surfaces were underestimated in convexity. This pattern of data suggests that perceived convexity estimates of all conditions were constrained at around 1.0 (i.e., towards the appearance of a sphere). This trend might be explained by prior knowledge that objects with a circular bounding contour will tend to be spherical rather than flatter or elongated. Theoretically, 3D shape can be estimated from a 2D image in a potentially infinite number of ways, but the human visual system tends to adopt the most likely interpretation (Richards et al., 1987). It could also be that the objects that are compressed or elongated along the line of sight are viewed nongenerically. Thus, the observers assume isotropic spherical shape based on the likelihood, which may explain why observers tend to adopt percepts of thickness that are close to 1.0 when confronted with a circular 2D bounding contour and limited perspective information generated by material properties and/or the surrounding environment (Kunsberg & Zucker, 2014).

Why might purely refractive objects be perceived flatter than the objects with other material properties? It is known that the surface slant of transparent objects tends to be underestimated, and the result depends on a combination of transparent object properties and the surrounding environment (Schlüter & Faul, 2019). It is possible that other sources of information may be relevant (e.g., Shape from texture). Shape from texture suggests that the human visual system can estimate object shape using the distortion of textural flow across the surface. Indeed, it is known that these distortions are caused by surface shape and an object’s gloss (Todd & Thaler, 2010). Similar textural gradients are provided by surfaces with relief in the form of mesostructure, which also changes scale as a function of surface orientation relative to the observer. In our static viewing conditions, we found that judgments of shape were more consistent across material properties and light fields for bumpy surfaces, presumably due to the inherent textural cues to 3D shape.

It is possible that the visual system may infer the shape of objects by variations in the compression gradients in these distortions. Indeed, the human visual system can estimate an object’s surface curvature from the distortion of surface textures and can also estimate object shape from this curvature information (Fleming et al., 2004; Todd & Thaler, 2010). This relationship between perceived shape and the distortion field can even be used for devising computations for estimating the relative convexity of objects; however, these computations do not reveal the magnitude of an object’s true convexity (Sakai & Finkel, 1995). The degree of distortion of the refractive object’s surface appearance also depends on the RI (Fleming, Jäkel, et al., 2011; Kawabe & Kogovšek, 2017). Thus, it may be difficult to estimate the magnitude of the convexity based on surface appearance.

Consistent with the view that the distortion field contributes to shape estimates, we found that variation in local RMS contrast of the surface could account for perceived convexity. We provided some computational evidence to suggest that observers might use this information within the upper portion of the image. It is possible that information concerning the shape of the bounding contour is also used to supplement the upper gradient information to experience shape across the entire region of the surface’s image (Adelson et al., 2009).

Fortunately, purely refractive objects or purely specular objects are rare in nature. Most naturally occurring objects contain surface materials with different optical components, for example, diffuse reflection, specular reflection, and refraction components including subsurface scattering. Therefore, human object shape recognition may have better accuracy when cues from diffuse and specular reflectance are added to those of transparent materials.

## Conclusion

We explored some of the limits in human visual perception of shape in computer-generated objects with different simulated surface optics. Our results suggest that the shape of purely refractive objects tends to be strongly underestimated, compared with surfaces with specular reflectance.

## Supplemental Material

sj-pdf-1-ipe-10.1177_2041669520982317 - Supplemental material for The Effect of Material Properties on the Perceived Shape of Three-Dimensional Objects Click here for additional data file. Supplemental material, sj-pdf-1-ipe-10.1177_2041669520982317 for The Effect of Material Properties on the Perceived Shape of Three-Dimensional Objects by Masakazu Ohara, Juno Kim and Kowa Koida in i-Perception
