# Supplementary material for: The Effect of Material Properties on the Perceived Shape of Three-Dimensional Objects
Source: Iperception. 2020 Dec 26;11(6):2041669520982317. doi: 10.1177/2041669520982317 (PMC7768321; doi:10.1177/2041669520982317)
Supplement: sj-pdf-1-ipe-10.1177_2041669520982317 - Supplemental material for The Effect of Material Properties on the Perceived Shape of Three-Dimensional Objects [file sj-pdf-1-ipe-10.1177_2041669520982317.pdf]

Supplemental materials for

The effect of material properties on the perceived shape of 3D objects

Masakazu Ohara[1,2], Juno Kim[2], Kowa Koida[1,3]

[1]*Department of Computer Science and Engineering, Toyohashi University of Technology, Japan*

[2]*School of Optometry and Vision Science, University of New South Wales, Australia*

[3]*Electronics-Inspired Interdisciplinary Research Institute, Toyohashi University of Technology, Japan*

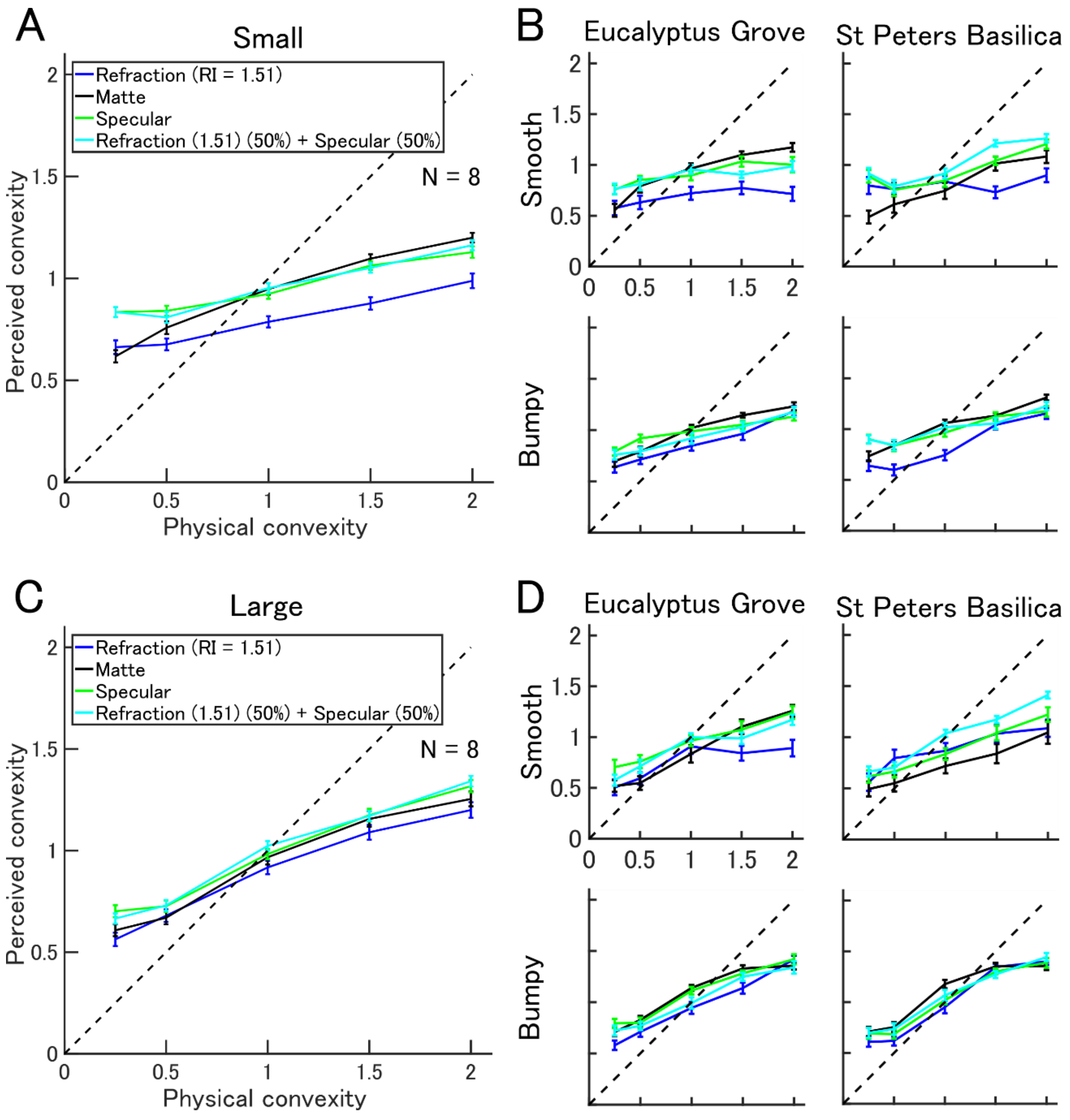

Figure S1 Effect of material properties on perceived convexity for different size stimuli. Format is same as in figure 3. (A, B) Results for the small object of ×0.5 size. (C, D) Results for the large object ×2.0 size.

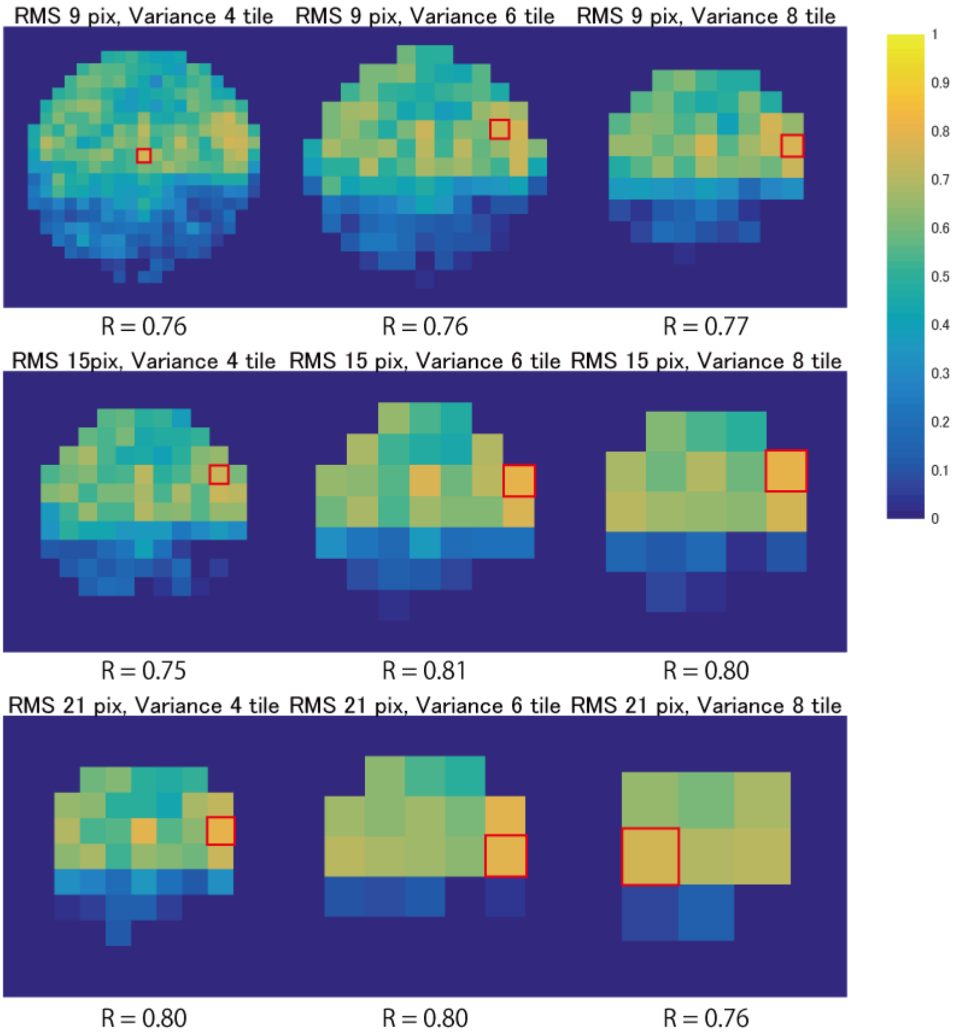

Figure S2 Correlation between 'local' RMS contrast variability and perceived convexity in various RMS pixel and variance tile size. These heat map were computed in the same way as figure 4E in main text except for the region size. Local RMS contrast was calculated in three different size (9 × 9, 15 × 15, 21 × 21 pixels; 0.61 × 0.61, 1.0 × 1.0, 1.4 × 1.4 deg, respectively), and variance of RMS contrast were calculated in three different regions (4 × 4, 6 × 6, 8 × 8). Red tiles show the area of highest correlation coefficient within each image. The R value on bottom shows the correlation coefficient in red square. As shown in main text, higher correlation was obtained for upper half of the image for all analysis.

Table S1. Statistics of experimental results (Figure 2)

This table show the result of 4-way (light probe, bump level, physical convexity and material property) anova.

Source	Sum of squares	d.f.	Mean squares	F	p	Partial η^2
Light probe	0.158	1	0.158	2.04	0.153	0.001
Bumpiness	0.037	1	0.037	0.48	0.489	0.000
Material	3.933	3	1.311	16.91	<0.001	0.026
Convexity	112.028	1	112.028	1445.23	<0.001	0.434
Light probe*Bumpiness	0.119	1	0.119	1.53	0.216	0.001
Light probe*Material	0.383	3	0.128	1.65	0.177	0.003
Light probe*Convexity	0.837	1	0.837	10.8	<0.001	0.006
Bumpiness*Material	2.333	3	0.778	10.03	<0.001	0.016
Bumpiness*Convexity	2.924	1	2.924	37.73	<0.001	0.020
Material*Convexity	0.633	3	0.211	2.72	0.043	0.004
Light probe*Bumpiness*Material	1.027	3	0.342	4.42	0.004	0.007
Light probe*Bumpiness*Convexity	0.018	1	0.018	0.24	0.627	0.000
Light probe*Material*Convexity	0.160	3	0.053	0.69	0.560	0.001
Bumpiness*Material*Convexity	2.380	3	0.793	10.23	<0.001	0.016
Light probe*Bumpiness*Material*Convexity	0.478	3	0.159	2.06	0.104	0.003
Error	146.349	1888	0.078			
Total	289.030	1919				

Post-hoc test: Tukey HSD (Refraction (RI = 1.51) vs other 3 surface materials)

Fig2A
p (vs matte) = <0.001
p (vs specular) = <0.001
p (vs refraction (50%) + specular (50%)) = <0.001
Fig2B
**Eucalyptus Grove - smooth**
p (vs matte) = <0.001
p (vs specular) = <0.001
p (vs refraction (50%) + specular (50%)) = <0.001
**Eucalyptus Grove - Bumpy**
p (vs matte) = 0.017
p (vs specular) = <0.001
p (vs refraction (50%) + specular (50%)) = 0.896
**St Peters Basilica - smooth**
p (vs matte) = 0.949
p (vs specular) = 0.193
p (vs refraction (50%) + specular (50%)) = <0.001
**St Peters Basilica - Bumpy**
p (vs matte) = 0.023
p (vs specular) = 0.149
p (vs refraction (50%) + specular (50%)) = 0.109

Table S2 Statistics of experimental results (Fig. S1)

This table show the result of 5-way (Object size (Large, small), light probe, bump level, physical convexity and material property) anova.

Source	Sum of squares	d.f.	Mean squares	F	p	Partial η^2
Size	4.364	1	4.364	59.340	<0.001	0.015
Light probe	0.045	1	0.045	0.620	0.433	0.000
Bumpiness	0.391	1	0.391	5.310	0.021	0.001
Material	3.717	3	1.239	16.850	<0.001	0.013
Convexity	147.132	1	147.132	2000.470	<0.001	0.346
Size*Light probe	0.116	1	0.116	1.580	0.209	0.000
Size*Bumpiness	0.157	1	0.157	2.140	0.144	0.001
Size*Material	0.698	3	0.233	3.160	0.024	0.003
Size*Convexity	9.818	1	9.818	133.480	<0.001	0.034
Light probe*Bumpiness	0.004	1	0.004	0.050	0.816	0.000
Light probe*Material	0.400	3	0.133	1.810	0.143	0.001
Light probe*Convexity	0.361	1	0.361	4.910	0.027	0.001
Bumpiness*Material	3.795	3	1.265	17.200	<0.001	0.013
Bumpiness*Convexity	2.629	1	2.629	35.740	<0.001	0.009
Material*Convexity	1.762	3	0.587	7.980	<0.001	0.006
Size*Light probe*Bumpiness	0.087	1	0.087	1.190	0.276	0.000
Size*Light probe*Material	0.270	3	0.090	1.220	0.300	0.001
Size*Light probe*Convexity	0.012	1	0.012	0.160	0.692	0.000
Size*Bumpiness*Material	0.089	3	0.030	0.400	0.750	0.000
Size*Bumpiness*Convexity	0.104	1	0.104	1.410	0.235	0.000
Size*Material*Convexity	1.136	3	0.379	5.150	0.002	0.004
Light probe*Bumpiness*Material	0.556	3	0.185	2.520	0.056	0.002
Light probe*Bumpiness*Convexity	0.003	1	0.003	0.050	0.830	0.000
Light probe*Material*Convexity	0.510	3	0.170	2.310	0.074	0.002
Bumpiness*Material*Convexity	3.761	3	1.254	17.050	<0.001	0.013
Size*Light probe*Bumpiness*Material	0.360	3	0.120	1.630	0.180	0.001
Size*Light probe*Bumpiness*Convexity	0.138	1	0.138	1.880	0.171	0.000
Size*Light probe*Material*Convexity	0.262	3	0.087	1.190	0.313	0.001
Size*Bumpiness*Material*Convexity	0.133	3	0.044	0.600	0.612	0.000
Light probe*Bumpiness*Material*Convexity	0.526	3	0.175	2.380	0.068	0.002
Size*Light probe*Bumpiness*Material*Convexity	0.266	3	0.089	1.210	0.306	0.001
Error	277.721	3776	0.074			
Total	485.801	3839				

Post-hoc test: Tukey HSD (Refraction (RI = 1.51) vs other 3 surface materials)

FigS1A
p (vs matte) = <0.001
p (vs specular) = <0.001
p (vs refraction (50%) + specular (50%)) = <0.001
FigS1B
**Eucalyptus Grove - smooth**
p (vs matte) = <0.001
p (vs specular) = <0.001
p (vs refraction (50%) + specular (50%)) = <0.001
**Eucalyptus Grove - Bumpy**
p (vs matte) = 0.359
p (vs specular) = 0.368
p (vs refraction (50%) + specular (50%)) = 0.992
**St Peters Basilica - smooth**
p (vs matte) = 1
p (vs specular) = 0.018
p (vs refraction (50%) + specular (50%)) = <0.001
**St Peters Basilica - Bumpy**
p (vs matte) = <0.001
p (vs specular) = <0.001
p (vs refraction (50%) + specular (50%)) = <0.001

FigS1C
p (vs matte) = 0.269
p (vs specular) = <0.001
p (vs refraction (50%) + specular (50%)) = <0.001

FigS1D
**Eucalyptus Grove - smooth**
p (vs matte) = 0.433
p (vs specular) = <0.001
p (vs refraction (50%) + specular (50%)) = 0.019
**Eucalyptus Grove - Bumpy**
p (vs matte) = 0.233
p (vs specular) = 0.118
p (vs refraction (50%) + specular (50%)) = 1
**St Peters Basilica - smooth**
p (vs matte) = 0.024
p (vs specular) = 1
p (vs refraction (50%) + specular (50%)) = 0.068
**St Peters Basilica - Bumpy**
p (vs matte) = 0.845
p (vs specular) = 1
p (vs refraction (50%) + specular (50%)) = 0.999

Post-hoc test: Tukey HSD (flattest (convexity = 0.25) matte object vs flattest specular object)

	p value
FigS1A	<0.001
FigS1B	
Eucalyptus Grove - smooth	1
Eucalyptus Grove - Bumpy	1
St Peters Basilica - smooth	0.003
St Peters Basilica - Bumpy	1
FigS1C	0.923
FigS1D	
Eucalyptus Grove - smooth	1
Eucalyptus Grove - Bumpy	1
St Peters Basilica - smooth	1
St Peters Basilica - Bumpy	1

Table S3 Statistics of experimental results (Fig. 3)

This table show the result of 5-way (Object position (Center, Left/Light), light probe, bump level, physical convexity and material property) anova.

Source	Sum of squares	d.f.	Mean squares	F	p	Partial η^2
Position	0.075	1	0.075	0.870	0.350	<0.001
Light probe	1.699	1	1.699	19.690	<0.001	0.003
Bumpiness	2.166	1	2.166	25.100	<0.001	0.004
Material	17.438	3	5.813	67.370	<0.001	0.034
Convexity	36.215	1	36.215	419.750	<0.001	0.069
Position*Light probe	0.012	1	0.012	0.140	0.709	<0.001
Position*Bumpiness	0.194	1	0.194	2.250	0.133	<0.001
Position*Material	0.106	3	0.035	0.410	0.746	<0.001
Position*Convexity	1.805	1	1.805	20.930	<0.001	0.004
Light probe*Bumpiness	0.146	1	0.146	1.690	0.193	<0.001
Light probe*Material	3.194	3	1.065	12.340	<0.001	0.006
Light probe*Convexity	1.443	1	1.443	16.720	<0.001	0.003
Bumpiness*Material	7.450	3	2.483	28.780	<0.001	0.015
Bumpiness*Convexity	0.490	1	0.490	5.680	0.017	<0.001
Material*Convexity	2.968	3	0.989	11.470	<0.001	0.006
Position*Light probe*Bumpiness	0.005	1	0.005	0.050	0.815	<0.001
Position*Light probe*Material	0.145	3	0.048	0.560	0.640	<0.001
Position*Light probe*Convexity	0.208	1	0.208	2.410	0.120	<0.001
Position*Bumpiness*Material	0.251	3	0.084	0.970	0.406	<0.001
Position*Bumpiness*Convexity	0.004	1	0.004	0.040	0.836	<0.001
Position*Material*Convexity	0.219	3	0.073	0.850	0.467	<0.001
Light probe*Bumpiness*Material	0.515	3	0.172	1.990	0.113	<0.001
Light probe*Bumpiness*Convexity	0.077	1	0.078	0.900	0.343	<0.001
Light probe*Material*Convexity	0.519	3	0.173	2.000	0.111	<0.001
Bumpiness*Material*Convexity	4.002	3	1.334	15.460	<0.001	0.008
Position*Light probe*Bumpiness*Material	0.033	3	0.011	0.130	0.945	<0.001
Position*Light probe*Bumpiness*Convexity	0.008	1	0.008	0.100	0.755	<0.001
Position*Light probe*Material*Convexity	0.181	3	0.060	0.700	0.552	<0.001
Position*Bumpiness*Material*Convexity	0.311	3	0.104	1.200	0.307	<0.001
Light probe*Bumpiness*Material*Convexity	0.668	3	0.223	2.580	0.052	<0.001
Position*Light probe*Bumpiness*Material*Convexity	0.126	3	0.042	0.490	0.692	<0.001
Error	491.436	5696	0.086			
Total	654.316	5759				

Post-hoc test: Tukey HSD (Refraction (RI = 1.51) vs other 3 surface materials)

Fig3A
p (vs matte) = 1
p (vs specular) < 0.001
p (vs refraction (50%) + specular (50%)) < 0.001
Fig3B
**Eucalyptus Grove - smooth**
p (vs matte) = 1
p (vs specular) < 0.001
p (vs refraction (50%) + specular (50%)) < 0.001
**Eucalyptus Grove - Bumpy**
p (vs matte) = 0.005
p (vs specular) < 0.001
p (vs refraction (50%) + specular (50%)) = 0.251
**St Peters Basilica - smooth**
p (vs matte) < 0.001
p (vs specular) < 0.001
p (vs refraction (50%) + specular (50%)) < 0.001
**St Peters Basilica - Bumpy**
p (vs matte) = 0.957
p (vs specular) = 1
p (vs refraction (50%) + specular (50%)) = 0.783

Fig3C
p (vs matte) = 1
p (vs specular) < 0.001
p (vs refraction (50%) + specular (50%)) < 0.001
Fig3D
**Eucalyptus Grove - smooth**
p (vs matte) = 0.420
p (vs specular) = < 0.001
p (vs refraction (50%) + specular (50%)) = < 0.001
**Eucalyptus Grove - Bumpy**
p (vs matte) = 0.002
p (vs specular) < 0.001
p (vs refraction (50%) + specular (50%)) = 0.567
**St Peters Basilica - smooth**
p (vs matte) < 0.001
p (vs specular) < 0.001
p (vs refraction (50%) + specular (50%)) < 0.001
**St Peters Basilica - Bumpy**
p (vs matte) = 1
p (vs specular) = 1
p (vs refraction (50%) + specular (50%)) = 1

Post-hoc test: Tukey HSD (Matte vs other 3 surface materials)

Fig3A
p (vs refraction (RI = 1.51)) = 1
p (vs specular) < 0.001
p (vs refraction (50%) + specular (50%)) < 0.001
Fig3B
**Eucalyptus Grove - smooth**
p (vs refraction (RI = 1.51)) = 1
p (vs specular) < 0.001
p (vs refraction (50%) + specular (50%)) < 0.001
**Eucalyptus Grove - Bumpy**
p (vs refraction (RI = 1.51)) = 0.005
p (vs specular) = 0.457
p (vs refraction (50%) + specular (50%)) = 1
**St Peters Basilica - smooth**
p (vs refraction (RI = 1.51)) < 0.001
p (vs specular) < 0.001
p (vs refraction (50%) + specular (50%)) < 0.001
**St Peters Basilica - Bumpy**
p (vs refraction (RI = 1.51)) = 0.957
p (vs specular) = 1
p (vs refraction (50%) + specular (50%)) = 1

Fig3C
p (vs refraction (RI = 1.51)) = 1
p (vs specular) < 0.001
p (vs refraction (50%) + specular (50%)) < 0.001

Fig3D
**Eucalyptus Grove - smooth**
p (vs refraction (RI = 1.51)) = 0.420
p (vs specular) < 0.001
p (vs refraction (50%) + specular (50%)) < 0.001
**Eucalyptus Grove - Bumpy**
p (vs refraction (RI = 1.51)) = 0.002
p (vs specular) = 0.639
p (vs refraction (50%) + specular (50%)) = 0.998
**St Peters Basilica - smooth**
p (vs refraction (RI = 1.51)) < 0.001
p (vs specular) < 0.001
p (vs refraction (50%) + specular (50%)) < 0.001
**St Peters Basilica - Bumpy**
p (vs refraction (RI = 1.51)) = 1
p (vs specular) = 1
p (vs refraction (50%) + specular (50%)) = 1
